# Comparison of 2-Year Outcomes between Intravitreal Ranibizumab and Intravitreal Aflibercept for Diabetic Macular Edema with “Treat-and-Extend” Regimen—Its Usefulness and Problems

**DOI:** 10.3390/jcm9092848

**Published:** 2020-09-02

**Authors:** Shinichiro Chujo, Masahiko Sugimoto, Taku Sasaki, Yoshitsugu Matsui, Kumiko Kato, Atsushi Ichio, Ryohei Miyata, Hisashi Matsubara, Mineo Kondo

**Affiliations:** Department of Ophthalmology, Mie University Graduate School of Medicine, 2-174 Edobashi, Tsu 514-8507, Japan; shinchujo21@gmail.com (S.C.); caesium1860@gmail.com (T.S.); footboyslim366@gmail.com (Y.M.); cqw14171jp@yahoo.co.jp (K.K.); atsushi_ichio@yahoo.co.jp (A.I.); ryohei.myt@gmail.com (R.M.); hmatsu@clin.medic.mie-u.ac.jp (H.M.); mineo@clin.medic.mie-u.ac.jp (M.K.)

**Keywords:** aflibercept, diabetic macular edema, ranibizumab, treat-and-extend regimen

## Abstract

Background: To compare the effectiveness of intravitreal ranibizumab (IVR) and intravitreal aflibercept (IVA) performed with the treat-and-extend (TAE) regimen on eyes with diabetic macular edema (DME). Patients and methods: This is a retrospective study of 125 eyes of 125 treatment-naïve DME patients who received anti-VEGF injections at three consecutive monthly intervals as the loading phase. The changes in the best-corrected visual acuity (BCVA), central retinal thickness (CRT), diabetic retinopathy severity scale (DRSS), and total injection numbers were compared between the two anti-VEGF agents. Results: Among 125 eyes, 26 eyes completed the treatment with the TAE regimen for 24 months (20.8%). Thirteen eyes of 13 patients (mean age, 70.9 ± 6.0 years) received intravitreal injections of 0.5 mg ranibizumab, and 13 eyes of 13 patients (65.9 ± 8.6 years) received 2 mg aflibercept. No significant differences were detected in the baseline demographics. At 24 months, BCVA was significantly improved in both groups; from 0.31 ± 0.19 to 0.10 ± 0.12 logMAR units for IVR and 0.41 ± 0.19 to 0.16 ± 0.28 logMAR units for IVA (*p* = 1.29 × 10^−9^). CRT was significantly reduced in both groups; 440.9 ± 69.3 to 307.5 ± 66.4 μm for IVR and 473.9 ± 71.5 to 317.8 ± 71.2 μm for IVA (*p* = 3.55 × 10^−9^). No significant differences were detected in the improvements of BCVA, CRT in both groups, and the total injection numbers for 24 months (11.0 ± 1.2 for the IVA group and 12.0 ± 1.0 the IVR group). DRSS was significantly improved in both groups (*p* = 0.0004 for IVR and *p* = 0.009 for IVA). Conclusion: No significant differences were detected in the improvements of BCVA or CRT and injection numbers between the IVR and IVA groups treated with the TAE regimen. These results indicate that the results of the treatment with both agents with the TAE regimen were equally effective, but only 20.8% of patients completed 24 months of continuous treatment with the TAE regimen. **Synopsis:** There are no significant differences regarding effectiveness between the IVR and IVA groups treated with the TAE regimen for DME eyes.

## 1. Introduction

Diabetic macular edema (DME) is a leading cause of blindness in working-age adults [1,2], and many factors are related to its development. It has been determined that the level of vascular endothelial growth factor (VEGF) is associated with the degree of vascular proliferation and hyperpermeability, and its suppression is effective in resolving DME [3]. Many randomized controlled trials have shown that intravitreal anti-VEGF injection is an effective first-line treatment for DME but required repeat injections and frequent visits [4,5,6,7,8]. Therefore, other flexible regimens are often used, and because of the risk–benefit balance, the optimal regimen has not been definitively established. Among them, treatment as needed, or the pro re nata (PRN) regimen, is frequently used. In fact, 75.0% of Japanese ophthalmologists prefer the PRN regimen for DME during the maintenance phase [9]. However, the PRN regimen is inconvenient because it requires frequent visits to determine the status of DME. In addition, the HORIZON trial for AMD found that nonadherence to the monthly monitoring led to a loss of the benefits of the previous treatments [10].

Much attention has been recently given to the treat-and-extend (TAE) regimen; its main goal is to minimize the number of injections and patient visits. The TAE regimen consists of a loading phase and a maintenance phase in which the injection interval is determined by the disease activity. Many studies have reported the effectiveness of the TAE regimen for AMD [11,12,13,14,15]. A meta-analysis of 26,360 patients from 42 real-world observational studies of intravitreal ranibizumab (IVR) showed that the TAE regimen resulted in better visual outcomes, with fewer visits compared to the PRN regimen for AMD at 2 years [16]. These results indicate that an individualized treatment and follow-up schedule is possible for each patient with the TAE regimen. Relevant to this study, the TAE regimen has also been reported to be effective for DME [17,18,19]. However, there has been only one small case series study that reported similar BCVAs after an optical coherence tomography (OCT)-guided TAE protocol and a visual acuity-guided PRN regimen [20]. Hence, it is still difficult to define the best treatment regimen for DME.

At present, three anti-VEGF agents, bevacizumab (Avastin^®^, Genentech, South San Francisco, CA, USA), ranibizumab (Lucentis^®^, Genentech, South San Francisco, CA, USA), and aflibercept (Eylea^®^, Regeneron Pharmaceuticals, Tarrytown, NY, USA), have been used to treat DME, and the former two agents have been approved for DME treatment in Japan. The Diabetic Retinopathy Clinical Research Network (DRCR.net) protocol-T study reported that aflibercept was more effective, especially in patients with initially poor vision, as long as a strict PRN regimen was followed [21]. However, there is no study that compares the effectiveness of IVR and intravitreal aflibercept (IVA) treatment with the TAE regimen for DME. Thus, the purpose of this study is to compare the effectiveness of IVR to that of IVA applied with the TAE regimen.

## 2. Experimental Section

### 2.1. Patients and Methods

This is a retrospective, single-center, comparative case series study. The procedures used in this study were approved by the Institutional Ethics Review Board of the Mie University Hospital (#702), and the study was registered at http://www.umin.ac.jp (UMIN ID 000033728).

**Figure 1:**
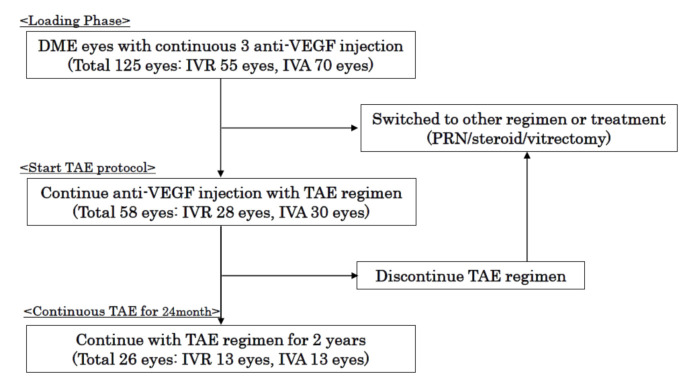
Flowchart of the study. A total of 125 eyes from 125 patients (55 patients received intravitreal ranibizumab (IVR) and 70 patients received intravitreal aflibercept (IVA)), diagnosed as treatment-naïve diabetic macular edema (DME), who had received 3-consecutive monthly IVR or IVA injections were registered. Among them, 58 eyes of 58 patients (46.4%, 28 patients received IVR and 30 patients received IVA) had injections with the treat-and-extend (TAE) regimen during the 2-year maintenance period. In the end, 26 eyes of 26 patients (20.8%, 13 patients received IVR and 13 patients received IVA) completed the 2-year treatment with the TAE regimen.

The medical charts of 125 eyes of 125 treatment-naïve DME patients in the database of Mie University Hospital were examined. The patients had been examined between April 2014 to November 2018. Of the 125 eyes, 55 eyes received IVR and 70 eyes received IVA. The patients received 3 consecutive IVR or IVA injections as a loading phase. Among them, 58 eyes of 58 patients were treated with the TAE regimen. In the end, 26 eyes of 26 patients completed the 24-month treatment with TAE regimen (Figure 1).

Each patient had a comprehensive ophthalmological examination, including measurements of the best-corrected visual acuity (BCVA) logMAR units and intraocular pressure, examination of the anterior segment by slit-lamp biomicroscopy, examination of the fundus by indirect ophthalmoscopy, and spectral-domain optical coherence tomography (SD-OCT) to determine the macular structure. 

Based on the fundus examination by indirect ophthalmoscopy by retina specialist (Shinichiro Chujo, Taku Sasaki, Yoshitsugu Matsui, Ryohei Miyata and Hisashi Matsubara), the severity of DR was classified into five categories: no DR, mild nonproliferative DR (mild NPDR), moderate NPDR, severe NPDR, and proliferative DR (PDR), according to the International Clinical Diabetic Retinopathy Disease Severity Scale (DRSS) [22].

The study inclusion criteria were (1) presence of center-involved DME diagnosed by clinical findings and fluorescein angiography, and CRT > 250 μm in the SD-OCT images at study entry, (2) age at least 20 years, and (3) BCVA pretreatment of ≥20/320. The exclusion criteria were prior ocular surgery, including cataract surgery within 6 months and during the experimental period, macular laser photocoagulation, and intravitreal or subtenon injections of steroids within 3 months of the beginning of the study. In addition, eyes with ocular inflammation, drusen, severe proliferative diabetic retinopathy, retinal hemorrhage that involved the intra- or subfoveal spaces, an epiretinal membrane, history of pars plana vitrectomy, glaucoma, and media opacities which affected the SD-OCT imaging, i.e., vitreous hemorrhage, vitreous opacity, severe cataract, and corneal opacity, were excluded. Patients with uncontrolled systemic conditions or a history of thromboembolic events were also excluded. The diabetes control was evaluated by the HbA1c levels (normal range: 4.6–6.2%), and renal dysfunction was evaluated by the estimated glomerular filtration rate (eGFR; normal range: 60–120 mL/min/m^2^).

After 3 consecutive monthly IVR or IVA injections as the loading phase, patients who did not respond to the anti-VEGF treatment (CRT remained ≥350 μm after treatment) or could not maintain the financial burden were excluded and were switched to other treatments, including steroid injection or vitrectomy. Patients who responded to IVR or IVA but did not care for the TAE regimen were switched to the PRN regimen and excluded from the study.

### 2.2. Intravitreal Injection of Anti-VEGF Agents

Intravitreal anti-VEGF injections were performed under local subconjunctival injection or topical anesthesia. Each patient received 0.5 mg of ranibizumab (IVR group) or 2 mg of aflibercept (IVA group) intravitreally with a 30-gauge needle that was inserted 4 mm posterior to the corneal limbus under sterile conditions. All patients received topical levofloxacin hydrate (1.5% Cravit ophthalmic solution^®^, Santen, Osaka, Japan) for 1 week after the injection.

### 2.3. Modified Treat and Extend (TAE) Regimen for DME

**Figure 2:**
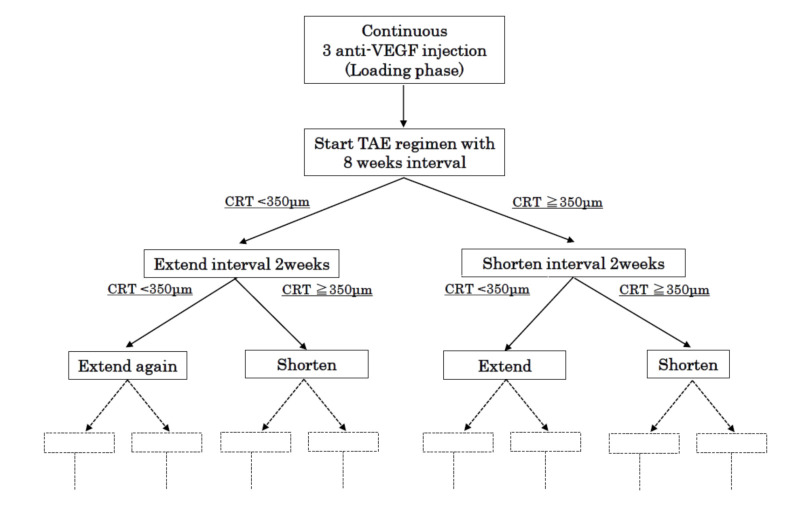
Modified-TAE regimen for diabetic macular edema (DME). All patients were given 3 consecutive monthly injections and they continued IVR or IVA with a modified TAE regimen. The initial treatment interval after the loading phase was set at 8 weeks. The injection interval was extended by 2 weeks if the central retinal thickness (CRT) was <350 μm at 2 consecutive examinations, and the injection interval was shortened by 2 weeks if CRT was ≥350 μm.

After 3 consecutive monthly injections of IVR or IVA, patients were treated with a modified TAE regimen, as shown in Figure 2. The first postloading phase injection interval was 8 weeks, then the injection interval was determined by the TAE protocol. The interval was extended by 2 weeks if the CRT was <350 μm at 2 consecutive examinations; alternatively, the injection interval was shortened by 2 weeks if the CRT was ≥350 μm. The minimum injection interval was set at 8 weeks. The maximum extended interval was not settled.

### 2.4. Measurements of Best-Corrected Visual Acuity (BCVA) 

BCVA was measured with a Landolt chart at every visit. The decimal BCVA was converted to the logarithm of the minimum angle of resolution (logMAR) units for the statistical analyses.

### 2.5. Optical Coherence Tomography (OCT)

The measurements of CRT were made on the images obtained by a Heidelberg Spectralis OCT instrument (Heidelberg Engineering Inc, Heidelberg, Germany). For qualitative and quantitative analyses of the SD-OCT images, the fast macula protocol was used to obtain the images with an automatic real-time mean value of 9, which acquired 25 horizontal lines consisting of 1024 A-scans per line. CRT was defined as the thickness between the internal limiting membrane and the retinal pigment epithelium at the fovea, and the value was automatically calculated from the center subfield of the macular thickness map using the bundled software.

### 2.6. Statistical Analyses

The results are presented as the means ± standard deviations (SDs). As the sample size was small and the statistical power is low, the Kolmogorov–Smirnov tests were used to determine the significance of the differences between corresponding pairs in the two groups. Two-way repeated-measures ANOVA and posthoc *t*-tests with Bonferroni’s corrections were used to determine the significance of the changes in BCVA and CRT. Chi-squared analysis was used to determine the significance of the differences in DRS between baseline and 24 months. Two-tailed *p* values of <0.05 were considered to be significant. The statistical evaluations were performed using the Statcel 4 statistical program (Statcel; OMC, Saitama, Japan).

## 3. Results

### 3.1. Clinical Characteristics of Patients at Baseline

The clinical characteristics of the patients are summarized in Table 1. Among 125 eyes, 58 eyes (46.4%) started anti-VEGF treatment with the TAE regimen. Finally, 26 eyes (20.8%) completed 24 months of continuous treatment with the TAE regimen. Among 26 eyes, 13 eyes of 13 DME patients received IVR, and 13 eyes of 13 DME patients received IVA. No significant differences in the baseline values were found between the IVR group and the IVA group; the mean age of the IVR group was 70.9 ± 6.0 years and that for the IVA group was 65.9 ± 8.6 years (*p* = 0.08), the HbA1c level was 7.6 ± 1.6% for the IVR group and 7.4 ± 1.8% for the IVA group (*p* = 0.33), and the eGFR level was 87.1 ± 16.1 mL/min/m^2^ for the IVR group and 71.0 ± 21.7 mL/min/m^2^ for the IVA group (*p* = 0.31). The mean baseline BCVA was 0.31 ± 0.19 logMAR units for the IVR group and 0.41 ± 0.19 logMAR units for the IVA group (*p* = 0.12, Table 1). The mean baseline CRT was 440.9 ± 69.3 μm for the IVR group and 473.9 ± 71.5 μm for the IVA group (*p* = 0.33, Table 1).

There were no ocular complications, including intraocular pressure elevations, infections, or adverse systemic events, that developed during the course of the experimental period for these 26 eyes.

### 3.2. Changes in DRSS during TAE Treatment Regimen

The results of the fundus examinations classified by DRSS are shown in Table 2. For the IVR group, the number of eyes classified as minimal nonproliferative diabetic retinopathy (NPDR) was 2, moderate NPDR was 8, and severe NPDR was 3. After 24 months of treatment, DRS was significantly improved (chi-squared analysis, *p* = 0.0004); the number of minimal NPDR was 12, moderate NPDR was 1, and severe NPDR was 0.

For the IVA group, the number of minimal NPDR was 5, moderate NPDR was 2, and severe NPDR was 6. After 24 months of treatment, DRS was significantly improved (chi-squared analysis, *p* = 0.009); the number of eyes with minimal NPDR was 12, moderate NPDR was 1, and severe NPDR was 0.

### 3.3. Changes in BCVA and CRT Following Treat-and-Extend Regimen

**Figure 3:**
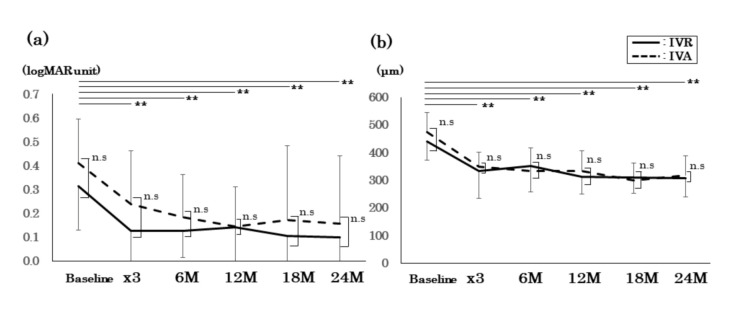
Changes in best-corrected visual acuity (BCVA, (**a**)) and central retinal thickness (CRT, (**b**)) following an intravitreal injection of ranibizumab (IVR) and aflibercept (IVA) applied with the treat-and-extend regimen. Two-way repeated-measures ANOVA and posthoc *t*-tests with Bonferroni’s corrections were used to determine the significance of the changes in BCVA and CRT. Kolmogorov–Smirnov tests were used to determine the significance of the differences between IVR and IVA. ** *p* < 0.01. Black line, IVR; broken line, IVA; n.s: no significant.

Significant BCVA improvement was observed during the period (0.10 ± 0.12 logMAR units at 24 months, with a *p*-value of 1.29 × 10^-9^ for the IVR group; 0.16 ± 0.28 logMAR units at 24 months, with a *p*-value of 1.31 × 10^−5^ for the IVA group). Significant CRT improvement was also observed during the period (307.5 ± 66.4 μm at 24 months, with a *p*-value of 3.55 × 10^−9^ for the IVR group; 317.8 ± 71.2 μm at 24 months, with a *p*-value of 2.00 × 10^−8^ for the IVA group) (Figure 3).

No significant differences were observed between the groups on all occasions with both BCVA and CRT (*p* > 0.05).

### 3.4. Number of Injections with TAE Regimen

**Figure 4:**
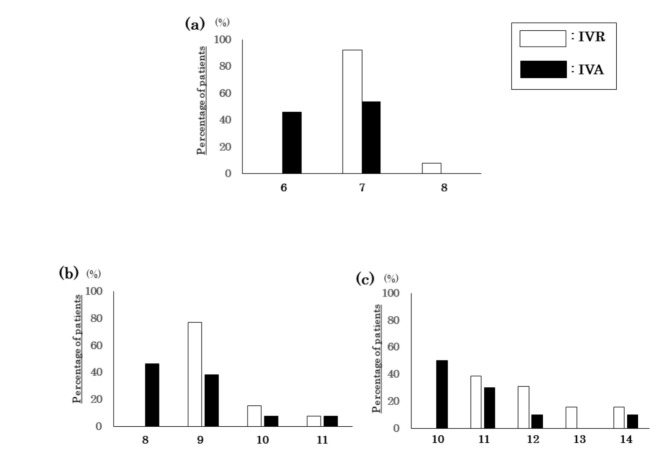
Number of intravitreal injections of ranibizumab (IVR) and aflibercept (IVA). Number of injections for each observation period is shown. The bar graph shows the number of injections at 12 months (**a**), 18 months (**b**), and 24 months (**c**). Data are shown as the percentage of all patients.

The mean number of injections at 12 months was 7.1 ± 0.3 times in the IVR group (median 7, range 7 to 8) and 6.5 ± 0.5 in the IVA group (median 7, range 6 to 7; *p* = 0.13, Kolmogorov–Smirnov tests, Figure 4a and Table 3). The mean injection number at 18 months was 9.5 ± 0.8 times in the IVR group (median 10, range 9 to 11) and 8.8 ± 0.9 in the IVA group (median 9, range 8 to 11, *p* = 0.13, Figure 4b and Table 3). The mean number of injections at 24 months was 12.0 ± 1.0 times in the IVR group (median 12, range 11 to 14) and 11.0 ± 1.2 in the IVA group (median 12, range 10 to 14, *p* = 0.29, Figure 4c and Table 3). The mean interval between injections at 24 months was 12.0 ± 3.4 weeks in the IVR group and 12.2 ± 3.2 weeks in the IVA group (*p* = 0.90). The mean maximum interval was 16 weeks in the IVR group and 16 weeks in the IVA group.

## 4. Discussion

Our results showed that there were no significant differences in BCVA, CRT, number of injections, and treatment intervals between IVR and IVA injections applied with the TAE regimen in eyes with DME. 

Many earlier studies have reported improvements in DR after multiple anti-VEGF injections. The subgroup analyses of the RIDE & RISE and the VIVID & VISTA studies showed improvements of DR in eyes with DME. These results showed the possibility that anti-VEGF agents can replace photocoagulation as a DR treatment [23,24,25,26]. Such improvements occurred due to the biological aspects of the anti-VEGF agents on the structural vascular features and the alterations of the hemodynamic properties [27]. Although these studies mentioned the effectiveness of anti-VEGF agents for DR improvement, they required many injections to obtain improvement. For example, Protocol-S required a mean of 14 injections (range 10 to 17) for a 2-year study period for eyes without DME at the baseline [23]. We also found an improvement of DR after 2-year treatment with the TAE regimen, and our injection numbers were fewer than that of other studies (12.0 ± 1.0 for the IVR group and 11.0 ± 1.2 for the IVA group). These differences indicate the possibility that DR improvement can be obtained with fewer injections with both IVR and IVA applied with the TAE regimen. Thus, the TAE regimen should be considered a good option for DR treatment.

For eyes with AMD, the TAE regimen required more injections than the PRN regimen but required fewer visits, with better visual outcomes [16]. When we compared our results to that of previous two-year studies on DME, including those with the TAE regimen, e.g., the RETAIN study and T-REX DME, our results are comparable to them even though the patient backgrounds and treatment protocol differed (Table 4). The changes in the mean BCVA and CRT with the TAE regimen appear to be comparable to that of the PRN regimen, as reported by the DRCR.net protocol-T. A fixed injection appears to deliver better improvements, but it required more injections than with the PRN or TAE regimen. On the other hand, the injection numbers were fewer with PRN, but the intervals were longer with the TAE regimen. The RETAIN study [17] compared the outcomes of the IVR–TAE regimen with the IVR–PRN regimen, and the results showed that the injection intervals could be extended to more than 8 weeks for 70% of the patients (9.2 weeks for TAE vs. 11.2 weeks for PRN). The T-REX DME study [19] compared the monthly IVR treatment group, the IVR–TAE group, and the IVR–TAE combined with laser group. The results of the T-REX study showed that significantly fewer injections were required for both the TAE groups than the monthly group (18.9 for TAE vs. 24.7 for monthly). In our study using the TAE regimen, the injection interval during the 24 months treatment period was longer than in previous studies, viz., 12.0 ± 3.4 weeks for IVR and 12.2 ± 3.2 weeks for IVA, and the maximum interval was 16.0 weeks for both agents, with no significant differences. These intervals were longer than that of the RETAIN study (9.2 weeks for IVR) and TREX-DME (6.2 weeks for IVR). The results of these earlier studies (Table 4) support our results, which indicates the effectiveness of the TAE regimen for DME compared to the as-needed PRN regimen.

There are many differences in the retreatment criterion among the different studies performed on eyes treated with the TAE regimen, i.e., the inactivity criteria, the extension length, and the strategy. Our study is a retrospective study, as opposed to the prospective RETAIN and TREX studies. In addition, we selected CRT of 350 µm as the main criterion to extend the interval between injections. This is comparable to the TREX-DME criterion of 325 μm [19]. However, the RETAIN study [17] based the extension criterion on visual acuity. It is known that a thinner CRT does not necessarily indicate a better BCVA because there are some cases where the outer segments of the photoreceptors are already damaged before DME can be resolved. This is called a paradoxical change between BCVA and CRT for DME [28]. As we defined CRT as a standard for treatment extension, this TAE regimen cannot cover those patients with lower CRT from poorer BCVA. In addition, another study has reported a similar visual acuity outcome between OCT-guided TAE protocol and visual-acuity-guided PRN regimen [20]. Thus, it is still not clear which criterion is the better one to use. Therefore, it was difficult to compare our results with those other studies. In addition, our TAE regimen used CRT as the only inactivity criterion. The resolution of intraretinal and subretinal fluids was not included in the criteria in our TAE regimen as with AMD management. Additionally, some studies reported no advantage of the TAE regimen compared to monthly or PRN regimens, although these were results from a small number of patients with a short duration. Ebneter et al. compared OCT-guided TAE to the visual-acuity-guided PRN regimen and found similar visual acuity outcomes [19]. Ehlers et al. reported similar visual acuity outcomes for the monthly and TAE regimen for persistent DME and switching from bevacizumab to ranibizumab [29]. Our result showed that only 26 of the 125 eyes (20.8%) could complete the 2 years of TAE protocol. This low ratio indicates the difficulty in completing the TAE regimen as a maintenance phase in the real world. We have previously reported the efficacy of the TAE protocol with intravitreal bevacizumab for managing DME [18]. Similar to this study, only 19.0% of patients could be treated with the TAE protocol and we concluded that the TAE protocol does not apply to every DME case. Other patients had to switch to other therapies. This is because there were nonresponders and we had to switch to other therapies, including steroid therapy or vitrectomy, to avoid irreversible damage to the outer segments of the photoreceptors induced by prolonged edema. In fact, many patients in our study switched to other therapies. We need more consideration for the disadvantages of TAE, although our results showed the effectiveness of both IVR and IVA with the TAE regimen. However, there is a possibility that once the protocol is properly fine-tuned, the TAE regimen may become a good treatment protocol for eyes with DME.

The DRCR.net protocol-T [21] study reported that IVA was more effective compared with IVR for eyes with poor baseline BCVA at one-year, but the difference was not significant at 2 years. There are several factors that can explain the differences between the two agents. Experiments on animals have shown that aflibercept penetrates deeper into the retina than ranibizumab after intravitreal injections in monkey eyes [30], and the VEGF concentration in the aqueous fluid was suppressed longer after IVA than IVR in monkey eyes [31]. In addition, aflibercept binds not only to VEGF and placenta growth factor but to galectin-1 [32]. From these different molecular characteristics of the two agents, there may exist different responses to the TAE regimen between IVR and IVA. Although the RETAIN and T-REX DME studies reported the usefulness of the TAE regimen for DME, none of the earlier studies have shown any differences between IVR and IVA with the TAE regimen. We also could not find any significant differences in BCVA, CRT, and the injection numbers between IVR and IVA. Similar to our result, Schwarzer et al. reported no significant differences in outcomes between IVR or IVA using an OCT-guided TAE protocol without a fixed loading phase [33]. There are some explanations for these differences. First, we did not divide the patients by BCVA at the baseline, which is different from the DRCR.net protocol-T study. Second, the DRCR.net protocol-T study reported significant differences only at 12 months and no significant difference at 24 months. Therefore, the molecular characteristics of the two agents may be subtle and may not be enough to cause differences as long as patients are treated with the TAE regimen.

There are some limitations to our study, including the small sample size. There is a possibility that our results did not have enough power to show any difference between the agents due to the small sample size. Second, in this study, 6 eyes of the 26 eyes (23.1%) could not extend the interval to more than 8 weeks. In the RETAIN study, about 30% of patients could not extend the interval to more than 8 weeks. These eyes received 8 weeks of fixed injections, and this is not a precise TAE regimen because it contributed less to the patients’ benefit. It is also important to consider other treatments for these cases, including sustained steroid agents [34], when the injection interval cannot be extended. Finally, we did not compare TAE results with the PRN regimen. Although the RETAIN study and T-REX DME reported the superiority of the TAE regimen compared to the PRN regimen, it is difficult to argue against the usefulness of the PRN regimen. In the real world, we mainly perform extra injections upon the complaints of the patients, without a strict PRN regimen. Hence, it is not clear whether a strict PRN regimen works better than the TAE regimen; this could be an area for further research.

## 5. Conclusions

No significant difference was observed between IVR and IVA with the TAE regimen. The TAE regimen, with both agents for DME, is a good option for patients with DME because it will result in good improvement of BCVA and CRT. However, only 20.8% of eyes could complete 24 months of continuous treatment with this regimen.

## Figures and Tables

**Table 1:** Patients’ backgrounds.

Agent	Age (Years)	HbA1c (%)	eGFR (ml/min/1.73m^2^)	BCVA (logMAR)	CRT (μm)
Ranibuzumab	70.9 ± 6.0	7.6 ± 1.6	87.1 ± 16.1	0.31 ± 0.19	440.9 ± 69.3
Aflibercept	65.9 ± 8.6	7.4 ± 1.8	71.0 ± 21.7	0.41 ± 0.19	473.9 ± 71.5
*p*-values	0.08	0.33	0.31	0.12	0.33

Data are the means ± standard deviations. Kolmogorov–Smirnov tests were used to determine the significance of the differences between the groups. BCVA, best-corrected visual acuity; CRT, central retinal thickness; eGFR, estimated glomerular filtration rate.

**Table 2:** Diabetic retinopathy severity evaluations.

DR Stage	Ranibuzumab		Aflibercept	
Baseline 24 months				
Minimum NPDR	2	12	5	12
Moderate NPDR	8	1	2	1
Severe NPDR	3	3	0	6
*p*-value	0.0004 **		0.009 **	

Chi-squared analysis was used to determine the significance of the differences between baseline and 24 months; ** *p* < 0.01.

**Table 3:** Injection numbers during maintenance phase.

Period	Ranibuzumab	Aflibercept	*p*-Value
12 months	7.1 ± 0.3	6.5 ± 0.5	0.13
18 months	9.5 ± 0.8	8.8 ± 0.9	0.13
24 months	12.0 ± 1.0	11.0 ± 1.2	0.29

Data are the means ± standard deviations. Kolmogorov–Smirnov tests were used to determine the significance of the differences between the groups.

**Table 4:** Comparisons of various DME treatment protocols.

Study	Regimen	VA Change (Letters)	CRT Change (μm)	Injection Numbers	Injection Interval (Patients Visit)
Protocol I ^#^	R PRN	+9.8	w.o laser −157	12	23 times/2 years ^###^
Protocol T (IVR)	R PRN	+12.3	−149	16	21.3 times/2 years ^###^
RETAIN (PRN)	R PRN	+8.1	−108 ^##^ (24.97%)	10.7	2.8 months (11.2 weeks)
T-REX (monthly)	R Fix	+7.5	−139	24.7	4.3 weeks
Protocol T (IVA)	A PRN	+12.8	−171	15	21.4 times/2 years ^###^
RETAIN (TAE)	R TAE	+6.5	w. o laser −113 ^##^	w. o laser (24.98%) 12.8 w. o laser	2.3 months (9.2 weeks)
T-REX (TAE)	R TAE	+9.6	w. o laser −140	w. o laser 18.9 w. o laser	6.2 weeks
This study (IVR)	R TAE	0.21	Log MAR −133.4	12.0	12.0 weeks
This study (IVA)	A TAE	0.23	Log MAR −140.5	11.0	12.2 weeks

(^#^) with deferred laser; (^##^) CRT changes were estimated from the percentage of changes from baseline; (^###^) injection intervals are not shown, and the total number of patient visits is shown. A, aflibercept; CRT, central retinal thickness; TAE, treat and extend; PRN, pro re nata; R, ranibizumab; VA, visual acuity; w.o laser, without laser treatment.

## Data Availability

The datasets used and/or analyzed during the current study are available from the corresponding author on reasonable request.
